# Competition and Integration of US Health Systems in the Post-COVID-19 New Normal: Cross-sectional Survey

**DOI:** 10.2196/32477

**Published:** 2022-03-24

**Authors:** Jiban Khuntia, Xue Ning, Rulon Stacey

**Affiliations:** 1 Business School University of Colorado Denver Denver, CO United States; 2 Business Department University of Wisconsin Parkside Kenosha, WI United States

**Keywords:** post-COVID-19, health system, competition, vertical integration, horizontal integration, COVID-19, integration, cross-sectional, survey, United States, characteristic, perception, decision

## Abstract

**Background:**

How do health systems in the United States view the concept of merger and acquisition (M&A) in a post-COVID 19 “new normal”? How do new entrants to the market and incumbents influence horizontal and vertical integration of health systems? Traditionally, it has been argued that M&A activity is designed to reduce inequities in the market, shift toward value-based care, or enhance the number and quality of health care offerings in a given market. However, the recent history of M&A activity has yielded fewer noble results. As might be expected, the smaller the geographical region in which M&A activity is pursued, the higher the likelihood that monopolistic tendencies will result.

**Objective:**

We focused on three types of competition perceptions, external environment uncertainty–related competition, technology disruption–driven competition, and customer service–driven competition, and two integration plans, vertical integration and horizontal integration. We examined (1) how health system characteristics help discern competition perceptions and integration decisions, and (2) how environment-, technology-, and service-driven competition aspects influence vertical and horizontal integration among US health systems in the post-COVID-19 new normal.

**Methods:**

We used data for this study collected through a consultant from a robust group of health system chief executive officers (CEOs) across the United States from February to March 2021. Among the 625 CEOs, 135 (21.6%) responded to our survey. We considered competition and integration aspects from the literature and ratified them via expert consensus. We collected secondary data from the Agency for Healthcare Research and Quality (AHRQ) Compendium of the US Health Systems, leading to a matched data set for 124 health systems. We used inferential statistical comparisons to assess differences across health systems regarding competition and integration, and we used ordered logit estimations to relate competition and integration.

**Results:**

Health systems generally have a high level of the four types of competition perceptions, with the greatest concern being technology disruption–driven competition rather than environment uncertainty–related competition and customer service–driven competition. The first set of estimation results showed that size, teaching status, revenue, and uncompensated care burden are the main contingent factors influencing the three competition perceptions. The second set of estimation results revealed the relationships between different competition perceptions and integration plans. For vertical integration, environment uncertainty–related competition had a significant positive influence (*P*<.001), while the influence of technology disruption–driven competition was significant but negative (*P*<.001). The influence of customer service–driven competition on vertical integration was not evident. For horizontal integration, the results were similar for environment uncertainty–related competition and technology disruption–driven competition; however, the significance of technology disruption–driven competition was weak (*P*=.05). The influence of customer service–driven competition in the combined model was significant and negative (*P*<.001).

**Conclusions:**

Competition-driven integration has subtle influences across health systems. Environment uncertainty–related competition is a significant factor, with underlying contingent factors such as revenue concerns and leadership as the leading causes of integration plans. However, technology disruption may hinder integrations. Undoubtedly, small- and low-revenue health systems facing a high level of competition are likely to merge to navigate the health care business successfully. This trend should be a focus of policy to avoid monopolistic markets.

## Introduction

### Background

The COVID-19 pandemic in 2020 transformed several aspects of the health care industry. Across the United States, health systems had to activate emergency plans, and cancel elective procedures, patient visits, and many nonessential activities, all while adopting remote and virtual communication care delivery models. As a result of the pandemic, providers have faced many new challenges and opportunities. Some of the financial and operational challenges have led to integrations among health systems to survive in the postpandemic “new normal,” with several health systems planning mergers and acquisitions (M&A) involving billions of dollars.

The rise in health care M&A is not entirely new; indeed, it has been increasing over the last decade, with total deals amounting to US $200 billion [1,2]. In 2020-2021 alone, several mergers worth billions of dollars have been in the spotlight. Recent announcements of UnitedHealth’s US $13 billion acquisition of Change Healthcare, Centene’s US $2.2 billion purchase of Magellan Health, Anthem’s deal for MMM Holdings in Puerto Rico, and Cigna’s acquisition of the urgent care telehealth provider MDLIVE exemplified both horizontal and vertical integration progressions in the US health care industry in 2021.

M&A reflects two underlying phenomena in the health care business: (1) competitive dynamics and struggle for survival, and (2) integration to solve competitive threats. This study focuses on relating competition perceptions and health systems’ integration plans, using data reported by chief executive officers (CEOs) in early 2021.

The objective of this study was two-fold. First, we sought to examine how health system characteristics lead to competition perceptions among health systems, as reported by CEOs in 2021. We assessed the differences between environment-, technology-, and service-driven competition perceptions in health systems with different characteristics, including size, region, ownership status, teaching status, revenue, number of physicians, and number of hospitals, among other factors.

The second objective of this study was to examine how these three types of competition perceptions influence vertical and horizontal integrations of US health systems in the post-COVID-19 new normal. Delineating competition perceptions and integration plans will help guide strategies and policies in health care.

### Competition Perceptions and Integration Plans

Beyond the recent disruptions due to COVID-19, health systems have been facing at least three types of competitive threats over the last decade, driving integrations more than ever before. First, uncertainties stemming from scientific developments have kept some health systems at the forefront of treatment, while others follow the developments [3]. For example, Detroit-based Henry Ford Health System and East Lansing–based Michigan State University partnered (as an integration, but not a merger) in 2020 to establish a fully integrated cancer program [4]. Similarly, fueled by expanding scientific horizons to care delivery, Atrium Health and Wake Forest Baptist Health merged to form a next-generation academic health system [5].

Second, technological developments have led to new solutions, which have led to new startups in the health care space, such as recently emerging remote and virtual care delivery firms and technology-enabled homecare delivery models (eg, DispatchHealth). The mergers of GigCapital2, UpHealth, and Cloudbreak Health as a unified telemedicine solution provider, and Teladoc’s acquisitions of several other technology-enabled models such as Livongo and InTouch Health, exemplify the competition and subsequent mergers due to technological imperatives [6].

Third, capturing a more significant share of patients’ care choices across the disease and life continuum increases revenue prospects. In other words, patient–service scope and scale have emerged to fuel competition among health systems. Salt Lake City–based Cimarron Healthcare’s acquisition of Ascent Behavioral Health Services, Monroe Capital, and Veronis Suhler Stevenson to expand into therapy and wellness areas is an example [7]. Similarly, the joint venture between Kindred Healthcare of Kentucky and Landmark Medical Center in Rhode Island (a subsidiary of Prime Healthcare Services from California) to own and operate a rehabilitation hospital showcases service expansion competition and integration imperatives [8].

Thus, attempts to adapt and change with innovation, while resisting specialized new generations of competitors on the one hand and the legacy burden of high-cost conglomerate structures to provide everything from high-level intensive care units to quick clinics colocated in drugstores as patient services on the other hand, have aggravated the competitive landscape for health systems [9]. Several very large health systems can bind or lock patients into their ecosystem, with a goal to emerge as a one-stop shop for the entirety of a patient’s health care needs and encounters “from the cradle to the grave,” in turn encouraging patients to think of the health system for all their health care needs [10]. Some of the examples we provide also reflect that competition has become more asymmetric and is expanding to organizations that are not in health care, such as technology, supply chain, or logistics firms [11].

Ignoring competition looming on the horizon will perish any health system, although managing the problem is not easy. Structural, operational, and strategic issues have only compounded the concerns. Health systems cannot move away from being all things to consumers. Competing in the health care business while lowering costs and improving efficiency may be possible, but only if health system leaders are innovative and proactive in integrating both high- and low-acuity care options into an effective strategy that seeks to capture a more significant share of patients’ health care service continuums and drive subsequent revenue opportunities. Two broad strategies help facilitate these goals: *vertical and horizontal integrations*.

*Vertical integration* expands the business by acquiring another company that operates before or after the acquiring company in the value chain. In contrast, horizontal integration is when a business grows by acquiring a similar company in their industry at the same value chain point [12]. Often, vertical integration is also done to increase network size and geographic coverage to mitigate risk in contracting, and to achieve market power over buyers and suppliers [13]. Small health systems or physician groups, which align with nonphysician partners such as hospitals, universities, medical schools, and health plans in health care, are touted as vertical integrations. In contrast, merging large or small health systems with similar expertise is an example of horizontal integration [14].

*Horizontal integration* might lead to enhanced operating efficiencies and economies of scale, improved coordination and quality of patient care, more in-house all-inclusive services, and higher-revenue services (eg, outpatient surgeries, imaging services) [15]. Through megamergers between large health systems, horizontal integration can thwart leaner niche competitors. Integration is challenging in any form because monitoring, coordination, and creating a cohesive culture across the integrated entities are always tricky. In addition, scope economies either do not exist or worsen the integrated organization’s dynamics.

The US health care industry has been characterized by different aspects of horizontal and vertical M&A. Such consolidations have changed the structure of US health care markets over time. Care integration has been an emerging solution to improve health quality and reduce costs, especially for a growing population of chronically ill patients in the United States [16]. More specifically, horizontal consolidation occurs when hospitals or physician groups merge, enabling the combined entity to increase its market share. Mergers reduce competing hospitals in a market, and vertical consolidations through acquisition of physician practices by a hospital reduce competition by reducing physicians vying for patients [17].

More structurally integrated organizations may manage care, coordinate across the care continuum, and exploit economies of scale and scope; such capabilities should, in theory, improve health care quality and lower costs. Practitioners and policymakers have therefore made investments in horizontal and vertical integrations. Vertical integration has been associated with better quality and is often framed as optimal care for specific conditions. Stakeholders perceive the positive impacts of consolidation on the long-term viability of health care facilities and their ability to adopt new care models, enhanced competition in health insurance, the creation of foundations, and pioneering medical research and innovation [18]. Stakeholders also believe that consolidation has changed geographic access to care; how physicians make referrals; and how educated patients are about care, the advertising environment, and economies in surrounding neighborhoods [19]. Market concentration also provides some benefits such as influencing utilization or readmissions and other potential health benefits [20].

Approaches to improve health system performance have been implemented to address care coordination problems and physician burnout. A widely advocated solution is the development of more integrated health delivery systems. While uncertainty remains about the drivers of organizational changes, these changes have led to health care organizations becoming more extensive and more financially integrated [21]. Horizontal and vertical integrations of hospitals and physicians have occurred rapidly for more than two decades. The COVID-19 pandemic may have exacerbated integration plans. Financial sustainability challenges because of declines in utilization and revenue emerged during COVID-19; the economic incentives for hospitals to merge, acquire physician practices, and employ more physicians are increasing. The financial and operational challenges faced during COVID-19 may incentivize health care organizations to make timely decisions for survival. Health systems might gain more significant market power through integration, resulting in greater competitive advantages [17].

### Theoretical Framework on Competition and Integration

Transaction cost economics theory has been applied in the health care context, and can plausibly explain the nuances associated with competition and integration dynamics [22]. Anchoring to the transaction costs perspective, this study proposes hazards exchange, transaction efficiency, and cost leadership as the three mechanisms behind the pathway from competition perceptions to integration plans. First, prior literature reveals that the transaction cost perspectives mostly have a focus on exchange hazards and can be used to explain the contracting between hospital groups [22], which forms the competition perceptions of a health system against another one, especially in uncertain environments. Second, transaction cost perspectives consider the efforts and costs required to complete the exchange and contracting activities [22]. Advanced information technologies such as cloud computing can significantly change the operation efficiency and cost, thus changing the economic paradigm and the relationship between collaborative parties [23]. This leads to the consideration of technology disruption–driven competition. Third, as health care is a service-based industry, the application of transaction cost perspectives in the health care context should consider the factor of customer service when perceiving the relationship with partners [24]. The competitive advantage framework proposed in extant research [25] considers three generic strategies: low-cost leadership, differentiation, and focus. This framework reveals the connection between transaction cost economics principles and competition categories, and highlights the significance of the target market or customer to sustain superior performance. This is consistent with customer service–driven competition. In other words, the needs of exchanging hazard in the external environment uncertainty–relevant competition results in integration, the efforts of improving transaction efficiency in the technology disruption–driven competition lead to integration, and the strategy of cost leadership in the customer service–driven competition causes integration.

More specifically, from the transaction cost economics perspective, vertical integration is about the economies of scope and horizontal integration is about the economies of scale [15]. In order to respond to the potential competition risks and change the competitive status, health systems are seeking either hospital-hospital mergers or hospital-physician integration [26]. Through the acquisitions of physician groups, health systems can expand the scientific horizons to care delivery [5]. Through the mergers of hospitals with similar focus, physicians in health systems can collaborate with similar expertise on a specific program [4]. Both integrations can reduce the hazards caused by uncertain environment–related competition. With higher perception, health systems will seek more unified solutions through vertical integration [6] and change the care delivery model through horizontal integration (eg, DisPatchHealth). The underlying mechanism for both cases is the improvement of transaction efficiency. However, such an outcome may cause even higher perception of technology-driven competition. Prior research suggests a positive relationship between CEO competition perceptions and integration, which arguably needs to be revisited given the recent post-COVID-19 “normal” contexts addressed in this study [27]. The perceptions of competition by CEOs of hospitals include the external market competition, and the integration considers the cost of information sharing. Strategic priorities in a health care system also propose the integrated framework of competition and collaboration, with discussions on competition around information technology [28]. Furthermore, the influence of customer service–driven competition on horizontal integration through the mechanism of cost leadership is reflected in the service niche [15]. The outcome of vertical integration when facing customer service–driven competition comes through the one-stop shop service to achieve cost leadership [10]. Details of these three mechanisms are summarized in Table 1.

**Table 1:** Mechanisms from competition perceptions to integration plans.

Transaction cost economics theory		Integration plans		
Competition reasons	Mechanisms		Vertical integration *(economies of scope)*	Horizontal integration *(economies of scale)*
Uncertain environment	Hazards exchange		Expansion	Collaboration
Technology disruption	Transaction efficiency		Unified solutions	Delivery models
Customer service	Cost leadership		One-stop shop	Service niche

## Methods

### Data Collection

The effort to assess the linkage between competition and integration prospects of health systems is part of a broad project undertaken by the Health Administration Research Consortium at the Business School of the University of Colorado Denver [29]. The project involves an annual and broad study on health systems and collects insights via a survey of health system CEOs. The insights will help policymakers, practitioners, and academic stakeholders as they collaborate to create strategies to help the industry respond to the pandemic and prepare for the next crisis.

A survey questionnaire was developed in December 2020 to collect data from health systems and to study the environment that health systems face scientifically. The survey items came from prior literature, with questions reworded to fit the health systems context. Inputs were taken from researchers, consultants, and executives with appropriate expertise to design the questions. The survey was validated using a scientific process of expert evaluations and was pilot-tested with five top executives from the Health Administration Program Advisory Board. The survey questionnaire was revised and finalized in January 2021.

A contact list of CEOs was compiled from 624 health systems across the United States using data from multiple sources, contacts, professional networks, websites, and annual reports. The survey instrument was implemented in a professional survey platform and was mapped emails to the platform to create unique, trackable links for each health system. Email and phone solicitations were made in multiple rounds between January 25 and March 2, 2021. A total of 148 responses from the 624 CEOs contacted, representing a 23.7% response rate, out of which 13 incomplete responses could not be used, leaving 135 final usable responses.

The 135 health systems represented in this survey varied from 1 to 18 hospitals with 176 to 75,000 employees. The annual revenue in 2020 of the health systems ranged from US $0.7 million to US $14 billion. The health systems aggregately represented US $300 billion in revenues and 1.1 million employees across the United States.

We then matched the survey data set with secondary data collected from the Agency for Healthcare Research and Quality (AHRQ) compendium to glean a complete picture of the health systems. Finally, we had data from 124 health systems located across the United States. We analyzed this combined data set, which yielded several important insights.

### Ethics Consideration

An ethics review was not applicable for this study. The data used was received through a leading professional consulting firm that anonymizes and provides secondary firm-level data for research and analysis to draw insights.

### Variables and Measures

Table 2 describes the variables used in this study. The main variables in this study are vertical integration (VINT), horizontal integration (HINT), external environment uncertainty–related competition (EEUC), technology disruption–driven competition (TDDC), and customer service–driven competition (CSDC). These variables were measured using 7-point Likert scales for relevant items. We tested the internal-consistency reliability of these multi-item variables using Cronbach *α*. The four *α* values were close to or greater than the recommended acceptable threshold of .70 for exploratory research.

**Table 2:** Description of variables, including survey questions and coding scheme.

Variable		Description
**Integration plans**		
	VINT^a^	Develop through vertical integration
	HINT^b^	Develop through horizontal integration
**Competition perceptions**		
	Main question	What is needed for your health system to compete in today’s post-COVID-19 economy?^c^
	EEUC^d^	External environment uncertainty–relevant competition (Cronbach *α*=.84); focus on services in which you excel, reevaluate the business you are in, anticipate policy issues and be ready for that
	TDDC^e^	Technology disruption–driven competition (Cronbach *α*=.73); transform through digital technologies, keeping current with technologies, new entrants disrupting the business model
	CSDC^f^	Customer service–driven competition (Cronbach *α*=.67); loyalty of customers, develop a mix of talent, quality of services, and patient satisfaction
**Contingent variables**		
	SIZE_B-SMALL, SIZE_B-MEDIUM, SIZE_B-LARGE	The three size variables of the health system are measured using the total beds managed by the health system across all hospitals, reported by the AHRQ^g^ Hospital Compendium: SIZE_B_SMALL, <100 beds; SIZE_B_MEDIUM, 100-400 beds; SIZE_B_LARGE, >400 beds
	REGION-NE, REGION-MW, REGION-SOUTH, REGION-WEST	The four region variables of the health systems are coded based on their primary location in the United States, following the Census Bureau categorization: REGION-NE, Northeast; REGION-MW, Midwest; REGION-SOUTH, South; REGION-WEST, West
	TEACHING-NON, TEACHING-MINOR, TEACHING-MAJOR	The three teaching variables are coded based on the teaching status of a health system: TEACHING-NON, nonteaching; TEACHING-MINOR, minor teaching; TEACHING-MAJOR, major teaching
	REVENUE-LOW, REVENUE-MEDIUM, REVENUE-HIGH	The three revenue variables of the health systems are measured using the health system’s annual revenue across all hospitals: REVENUE-LOW, <US $2 billion; REVENUE-MEDIUM, US $2-5 billion; REVENUE-HIGH, >US $5 billion
	HIGH-DSH-HOSP	The health system includes at least one high DSH^h^ patient percentage hospital (1=yes, 0=no)
	HIGH-BURDEN-SYS	Health system–wide uncompensated care burden flag (1=yes, 0=no)
	HIGH-BURDEN-HOSP	The health system includes at least one high uncompensated care burden hospital (1=yes, 0=no)
	OWNERSHIP	Predominantly investor-owned hospitals (1=yes, 0=no)
	PHYSICIANS	The number of physicians in the health system, measured by the number of physicians reported by the AHRQ Hospital Compendium
	HOSPITALS	Number of hospitals the health system has reported by the AHRQ Hospital Compendium

^a^VINT: vertical integration.

^b^HINT: horizontal integration.

^c^All questions are measured using a 7-point Likert scale; 1=strongly disagree to 7=strongly agree.

^d^EEUC: external environment uncertainty–related competition.

^e^TDDC: technology disruption–driven competition.

^f^CSDC: customer service–driven competition.

^g^AHRQ: Agency for Healthcare Research and Quality.

^h^DSH: discharge level.

The influencing factors examined in this study represent several categories: size, region, teaching status, revenue, and several other system characteristics. These variables are coded (see Table 2) to reflect the characteristics of a health system that may influence its competition perception and integration preference. Three size variables measure the number of beds across a health system (SIZE_B-SMALL, SIZE_B-MEDIUM, and SIZE_B-LARGE), four region variables reflect the location of a health system (REGION-NE, REGION-MW, REGION-SOUTH, and REGION-WEST), three teaching status–related variables assess the extent to which a health system is associated with a teaching program (TEACHING-NON, TEACHING-MINOR, and TEACHING-MAJOR). Three revenue variables measure the annual revenue of a health system (REVENUE-LOW, REVENUE-MEDIUM, REVENUE-HIGH). In addition, we included variables to capture the discharge levels of patients (HIGH-DSH-HOSP), uncompensated care burden (HIGH-BURDEN-SYS and HIGH-BURDEN-HOSP), ownership status (OWNERSHIP), number of physicians (PHYSICIANS), and number of hospitals (HOSPITALS).

### Sample Statistics

The descriptive statistics and pairwise correlations among the key variables used in this study appear in Table 3 and Multimedia Appendix 1, respectively. As shown in Table 3, health systems have a relatively high perception of TDDC compared with EEUC and CSDC. Furthermore, horizontal integration seems to be more popular for health systems than vertical integration.

In addition, to check for nonresponse bias, we compared the characteristics of responding and nonresponding health systems. Detailed comparisons are shown in Table 4. The *t* test results for all comparisons indicated no significant difference between respondents and nonrespondents.

**Table 3:** Summary statistics (N=124).

Variable^a^	Mean (SD)	Range
EEUC	5.10 (1.39)	1.67-7.00
TDDC	5.63 (0.94)	2.67-7.00
CSDC	5.12 (1.21)	2.00-6.67
VINT	4.51 (1.85)	1.00-7.00
HINT	4.97 (1.53)	1.00-7.00
SIZE_B-SMALL	0.09 (0.28)	0.00-1.00
SIZE_B-MEDIUM	0.37 (0.49)	0.00-1.00
SIZE_B-LARGE	0.54 (0.50)	0.00-1.00
REGION-NE	0.22 (0.42)	0.00-1.00
REGION-MW	0.24 (0.43)	0.00-1.00
REGION-SOUTH	0.35 (0.48)	0.00-1.00
REGION-WEST	0.18 (0.38)	0.00-1.00
TEACHING-NON	0.30 (0.46)	0.00-1.00
TEACHING-MINOR	0.48 (0.50)	0.00-1.00
TEACHING-MAJOR	0.22 (0.41)	0.00-1.00
REVENUE-LOW	0.61 (0.49)	0.00-1.00
REVENUE-MEDIUM	0.23 (0.43)	0.00-1.00
REVENUE-HIGH	0.15 (0.35)	0.00-1.00
HIGH-DSH-HOSP	0.33 (0.47)	0.00-1.00
HIGH-BURDEN-SYS	0.20 (0.40)	0.00-1.00
HIGH-BURDEN-HOSP	0.30 (0.46)	0.00-1.00
OWNSHIP	0.02 (0.13)	0.00-1.00
PHYSICIANS	1.84 (0.80)	1.00-3.00
HOSPITALS	1.50 (0.77)	1.00-3.00

^a^See Table 2 for a description of variable codes.

**Table 4:** Characteristics of responding and nonresponding health systems.

Characteristics^a^		Respondents (n=124), n (%)	Nonrespondents (n=511), n (%)	*t* test (*633*)
**Size**				
	Small (6-99 beds)	11 (8.9)	42 (8.2)	–0.19
	Medium (100-399 beds)	45 (36.3)	212 (41.3)	–0.56
	Large (≥400 beds)	68 (54.8)	257 (50.3)	1.41
**Region**				
	Northeast	27 (21.8)	117 (22.9)	0.07
	Midwest	30 (24.2)	133 (26.0)	0.55
	South	45 (36.3)	169 (33.1)	–0.48
	West	22 (17.7)	92 (18.0)	–0.12
**Physicians**				
	Small (51-199 physicians)	50 (40.3)	189 (37.0)	–0.74
	Medium (200-999 physicians)	41 (33.1)	204 (39.9)	–0.69
	Large (≥1000 physicians)	33 (26.6)	118 (23.1)	1.53
**Hospitals**				
	Small (1-3 hospitals)	83 (66.9)	337 (65.9)	–1.27
	Medium (4-6 hospitals)	20 (16.1)	67 (13.1)	–0.02
	Large (≥7 hospitals)	21 (16.9)	107 (20.9)	0.81
**Ownership status**				
	Investor-owned	3 (2.4)	15 (2.9)	–0.85
	Noninvestor-owned	121 (97.6)	496 (97.1)	0.85
**Teaching status**				
	Major teaching	29 (23.4)	138 (27.0)	–0.15
	Minor teaching	58 (46.8)	225 (44.0)	–0.61
	Nonteaching	37 (29.8)	148 (29.0)	0.85

^a^The number of physicians and hospitals are presented in this table in different categories for easy comparison across respondents and nonrespondents.

### Statistical Analysis

To answer the two research questions stated earlier, we performed two sets of analyses. We used ordered logit regressions to estimate (1) the relationship of the three competition perceptions to specific hospital characteristics and (2) the relationship between competition perceptions and integration plans. The integration variables are ordinal (with a sequentially higher value), thus driving the decision for ordered logit regressions. The ordered logit approach does not assume equal intervals between levels in the dependent variable and is thus a preferred estimation than ordinary least square estimation processes that assumes equal linear intervals. The ordered logit model is as follows:

Y_i_^*^=βX_i_+e_i_,

where Y_i_^*^ represents respondents’ propensity to indicate higher levels of the dependent variables (ie, EEUC, TDDC, CSDC, VINT, and HINT), *X_i_* is a set of explanatory variables, *β* is a vector of parameters, and *e_i_* are disturbances.

We did not observe Y_i_^*^. Instead, we observed the ordinal dependent variable *Y_i_* depending on the values of thresholds or cut-off points *τ_m_*_–1_ and *τ_m_*. The probability distribution of *Y_i_* is given as follows:

Pr(Y_i_=m|X_i_=F(τ_m_–Xβ)–F(τ_m__–1_–Xβ)

## Results

### Estimation Outcomes

Table 5 shows the results of the ordered logit model estimation that describe the relationship between contingent factors and each of the three types of competition perceptions.

One teaching variable had a significant, negative association with EEUC. The major teaching health systems variable showed high statistical significance (*P*<.001). This suggests that major teaching health systems tend to have lower perceptions of EEUC. Based on the marginal-effects analysis, we found a 1.1% decrease in the probability of perceiving EEUC among major teaching health systems compared with nonteaching health systems.

There was also a significant negative relationship between high revenue and EEUC (*P*<.001), indicating that high-revenue health systems tend to perceive less EEUC than low-revenue health systems. The marginal-effects analysis suggested a 0.71% decrease in the probability of perceiving EEUC among high-revenue health systems than low-revenue health systems.

A high-burden system had a significant positive impact on EEUC (*P*<.001), while a high-burden hospital had a significant negative impact. These results indicate that (1) a health system with a system-wide high uncompensated care burden tends to perceive EEUC, while (2) a health system with no high uncompensated care burden hospital is less likely to perceive EEUC. We also examined the marginal effects of these two variables. The results indicated a 0.52% increase in the probability of perceiving EEUC by a health system with a system-wide high uncompensated care burden and a 1.32% decrease by a health system with at least one high uncompensated care burden hospital.

For TDDC, there was a significant negative relationship between this perception and both medium size (*P*<.001) and large size (*P*=.04), indicating that smaller-sized health systems are more likely to have a higher level of TDDC. The marginal-effects analysis showed that the probability changes for these two factors are 3.08% and 2.13%, respectively.

Region had significant effects on the perception of TDDC. For example, health systems in the south (*P*<.001) have a higher TDDC perception than those in the northeast. The change in the marginal effects was 0.92%. Similarly, there was a significant positive relationship between high revenue and TDDC (*P*<.001). This result indicates that high-revenue health systems tend to perceive more TDDC than low-revenue health systems. The marginal effect of this change was 0.93%.

The relationship between a high system-wide burden and TDDC and the relationship between total hospitals in a health system and TDDC were both significant and negative (*P*<.001). These results suggest that health systems without a system-wide high uncompensated care burden and those with fewer hospitals are more likely to perceive TDDC. The marginal effects for these two variables were 1.14% and 0.59%, respectively.

For CSDC, compared with small-sized health systems (ie, those with a fewer number of beds), medium- and large-sized health systems (*P*<.001) are less likely to perceive CSDC. These results reveal the strong influence of health systems’ size on their competition perceptions regarding customer service provision. More specifically, marginal-effects analysis indicated a 1.11% and 0.49% decrease in the probability of perceiving CSDC for medium- and large-sized health systems, respectively.

**Table 5:** Influence of contingent factors from the ordered logit model estimation.^a^

Variables	EEUC^b^		TDDC^c^		CSDC^d^	
	Coefficient (SE)	*P* value	Coefficient (SE)	*P* value	Coefficient (SE)	*P* value
SIZE-MEDIUM	–.379 (.122)	.002	–1.789 (.184)	<.001	–1.468 (.124)	<.001
SIZE-LARGE	.463 (.358)	.20	–1.673 (.826)	.04	–.864 (.164)	<.001
REGION-MW	.046 (.261)	.86	.011 (.277)	.97	–.660 (.484)	.17
REGION-SOUTH	.292 (.099)	.003	.824 (.118)	<.001	.294 (.378)	.44
REGION-WEST	1.210 (.717)	.09	.210 (.570)	.71	.773 (.959)	.42
TEACHING-MINOR	–.575 (.548)	.29	.304 (.183)	.10	–.181 (.424)	.67
TEACHING-MAJOR	-1.348 (.068)	<.001	–.081 (.211)	.70	–.995 (.112)	<.001
REVENUE-MEDIUM	–.702 (.449)	.12	.932 (.359)	.009	–.217 (.054)	<.001
REVENUE-HIGH	–.961 (.162)	<.001	1.056 (.130)	<.001	–.200 (.106)	.06
HIGH-DSH-HOSP	.431 (.848)	.61	.639 (.282)	.02	.425 (.422)	.31
HIGH-BURDEN-SYS	1.434 (.056)	<.001	–.739 (.116)	<.001	.314 (.649)	.63
HIGH-BURDEN-HOSP	–1.668 (.129)	<.001	.241 (.169)	.15	–.977 (.311)	.002
OWNERSHIP	.333 (.552)	.55	–1.644 (1.337)	.22	–.667 (1.477)	.65
PHYSICIANS	.224 (.107)	.04	–.022 (.643)	.97	–.057 (.205)	.78
HOSPITALS	.004 (.028)	.88	–.482 (.125)	<.001	.221 (.130)	.09
Pseudo *R*^2^	0.057	—^e^	0.036	—	.043	—

^a^The results of the cut-off points are omitted for brevity.

^b^EEUC: environment uncertainty–related competition.

^c^TDDC: technology disruption–driven competition.

^d^CSDC: customer service–driven competition.

^e^not applicable.

There was a significant negative relationship between major teaching health systems and CSDC (*P*<.001), indicating that compared with nonteaching health systems, those with a teaching focus tend to perceive less CSDC. The marginal-effects analysis indicated a 0.78% decrease in the probability of perceiving CSDC among major teaching health systems than among nonteaching health systems.

In addition, there was a significant negative relationship between CSDC and medium revenue (*P*<.001), suggesting that health systems with a midrange revenue may not favor perceived CSDC. The marginal-effects analysis showed a 0.13% impact for this revenue variable.

Table 6 and Table 7 display the estimation results for the second set of models, illustrating the relationship between competition perceptions and vertical and horizontal integration, respectively. The results in Table 6 show different relationships between the three types of competition perceptions and vertical integration. Models 1-3 demonstrate the direct relationships of EEUC, TDDC, and CSDC with VINT, respectively, while model 4 is the combined model, which includes all three competition perceptions. The results indicated a significant positive relationship (*P*<.001) between EEUC and VINT, and a significant negative relationship (*P*<.001) between TDDC and VINT. The relationship between CSDC and VINT was not significant. More specifically, the results suggest that health systems with higher EEUC perceptions tend to choose vertical integration plans. The marginal-effects analysis showed a value of 4.64% for this variable. However, health systems with a higher level of TDDC are less likely to opt for vertical integration. The probability change, according to the marginal-effects analysis, was 4.11% for TDDC.

**Table 6:** Ordered logit model estimation results for competition types and vertical integration.^a^

Variables	Model 1		Model 2		Model 3		Model 4 (combined)	
	Coefficient (SE)	*P* value	Coefficient (SE)	*P* value	Coefficient (SE)	*P* value	Coefficient (SE)	*P* value
EEUC^b^	1.203 (.152)	<.001	—^c^	—	—	—	1.601 (.255)	<.001
TDDC^d^	—	—	–.515 (.132)	<.001	—	—	–1.029 (.162)	<.001
CSDC^e^	—	—	—	—	.163 (.183)	.37	-.111 (.161)	.49
SIZE	.228 (.666)	.73	.288 (.284)	.31	.371 (.518)	.47	.021 (.371)	.95
REGION	.451 (.205)	.03	.553 (.015)	<.001	.460 (.030)	<.001	.567 (.141)	<.001
OWNERSHIP	–.135 (3.608)	.97	.347 (2.488)	.89	.561 (3.942)	.89	–.612 (1.908)	.75
TEACHING	.648 (.587)	.27	–.053 (.493)	.92	.026 (.602)	.97	.739 (.606)	.22
REVENUE	–.105 (.042)	.01	–.288 (.035)	<.001	–.349 (.033)	<.001	.111 (.066)	.09
HIGH-DSH^f^-HOSP^g^	–.793 (.635)	.21	–.184 (.447)	.68	–.453 (.301)	.13	–.590 (.607)	.33
HIGH-BURDEN-SYS^h^	.149 (.958)	.88	1.002 (.434)	.02	.924 (.636)	.15	–.026 (.851)	.98
HIGH-BURDEN-HOSP	–.529 (.408)	.20	–1.365 (.178)	<.001	–1.087 (.354)	.002	–.560 (.303)	.07
PHYSICIANS	–.347 (.211)	.10	.010 (.176)	.95	–.035 (.160)	.83	–.282 (.437)	.52
HOSPITALS	.022 (.221)	.92	–.048 (.100)	.63	–.020 (.081)	.80	–.086 (.294)	.77
Pseudo *R*^2^	0.187	N/A^i^	0.069	N/A	.053	N/A	.254	N/A
Mean VIF^j^	1.89	N/A	1.73	N/A	1.73	N/A	2.07	N/A

^a^The results of the cut-off points are omitted for brevity.

^b^EEUC: environment uncertainty–related competition.

^c^Not included in model.

^d^TDDC: technology disruption–driven competition

^e^CSDC: customer service–driven competition.

^f^DSH: discharge.

^g^HOSP: hospital.

^h^SYS: system.

^i^N/A: not applicable.

^j^VIF: variance inflation factor.

Similarly, the results in Table 7 display the different direct (models 1, 2, and 3) and combined (model 4) effects of the three types of competition perceptions on horizontal integration. The most significant relationship was the impact of EEUC on HINT (*P*<.001). This significant and positive relationship is consistent in both the direct and combined models, indicating that health systems with higher EEUC perceptions have a higher probability of following horizontal integration plans. The negative relationship between TDDC and HINT was significant (*P*=.05) in the combined model. This result suggests a lower probability of adopting horizontal integration for health systems with higher TDDC perceptions. Finally, although the direct relationship between CSDC and HINT was not significant in the direct model, the combined model showed a highly significant negative relationship (*P*<.001) between these variables. This result indicates that health systems that perceive a higher level of CSDC are more likely to pursue a horizontal integration approach.

**Table 7:** Ordered logit model estimation results for competition types and horizontal integration.^a^

Variables	Model 1		Model 2		Model 3		Model 4 (combined)	
	Coefficient (SE)	*P* value	Coefficient (SE)	*P* value	Coefficient (SE)	*P* value	Coefficient (SE)	*P* value
EEUC^b^	.619 (.036)	<.001	—^c^	—	—	—	1.360 (.130)	<.001
TDDC^d^	—	—	–.655 (.336)	.05	—	—	–.219 (.111)	.05
CSDC^e^	—	—	—	—	–.309 (.289)	.28	–1.153 (.210)	<.001
SIZE	.395 (.422)	.35	.305 (.258)	.24	.423 (.443)	.34	.365 (.135)	.007
REGION	.199 (.230)	.39	.359 (.097)	<.001	.372 (.041)	<.001	.397 (.075)	<.001
OWNERSHIP	–.712 (1.913)	.71	–.666 (1.375)	.63	–.539 (1.543)	.73	–1.679 (.829)	.04
TEACHING	.152 (.696)	.83	–.041 (.774)	.96	–.135 (.817)	.87	.035 (.669)	.96
REVENUE	–.138 (.274)	.61	–.226 (.263)	.39	–.432 (.120)	<.001	–.120 (.373)	.75
HIGH-DSH^f^-HOSP^g^	–.653 (.317)	.04	–.334 (.227)	.14	–.375 (.255)	.14	–.474 (.223)	.03
HIGH-BURDEN-SYS^h^	.947 (.395)	.02	1.241 (.490)	.01	1.440 (.470)	.002	.740 (.648)	.25
HIGH-BURDEN-HOSP	–.199 (.513)	.70	–.710 (.454)	.12	–1.019 (.228)	<.001	–.548 (.378)	.15
PHYSICIANS	.177 (.165)	.28	.224 (.313)	.47	.310 (.184)	.09	.183 (.300)	.54
HOSPITALS	–.219 (.507)	.67	–.193 (.468)	.68	–.048 (.321)	.88	–.004 (.498)	.99
Pseudo *R*^2^	.102	N/A^i^	.080	N/A	.062	N/A	.212	N/A
Mean VIF^j^	1.78	N/A	1.73	N/A	1.74	N/A	2.00	N/A

^a^The results of the cut points are omitted for brevity.

^b^EEUC: environment uncertainty–related competition.

^c^Not included in model.

^d^TDDC: technology disruption–driven competition

^e^CSDC: customer service–driven competition.

^f^DSH: dispatch.

^g^HOSP: hospital.

^h^SYS: system.

^i^N/A: not applicable.

^j^VIF: variance inflation factor.

## Discussion

### Principal Findings

This study explored the differences of three competition perceptions of health systems in the United States, contingent on their characteristics. The study further examined how competition aspects are related to the different integration plans. The main findings are two-fold. For the first set of explorations on the main factors that influence health systems’ competition perceptions, asking about the similarities and differences of such influences and their implications, we found that size, teaching status, revenue, and burden are the four main factors influencing health systems’ competition perceptions.

First, the results indicate that small-sized health systems will perceive a higher level of all three types of competition perceptions. Therefore, a driving reason for integration plans among small-sized health systems may be to mitigate competition. The influences of size on all three competition perceptions were consistent.

Second, compared with nonteaching health systems, major teaching health systems perceive less EEUC and CSDC. A potential reason is an emphasis on major teaching health systems; they are less bothered by the external environment and customer service provision changes.

Third, the results indicate that revenue has opposite influences for TDDC perceptions and EEUC and CSDC perceptions. On the one hand, the results suggest that high-revenue health systems perceive a higher level of TDDC. On the other hand, health systems with more revenue (both medium and high revenue) perceive lower EEUC and CSDC. These results demonstrate the nuanced influences of revenue on competition perceptions, and imply that the role of revenue should be examined more carefully in relevant decision-making.

Fourth, according to prior research (AHRQ), there are two types of uncompensated care burdens: a system-wide burden and hospital-level burden. This study employed two variables, high-burden system and high-burden-hospital, to capture these two types of burdens. The results showed that they have different influences on different competition perceptions. For example, health systems with a system-wide burden are more likely to perceive EEUC, while those with at least one high-burden hospital are less likely to perceive EEUC.

For the second set of examinations, we found a consistent significant positive relationship between EEUC and both vertical and horizontal integration; a consistent negative relationship, with changing significance, between TDDC and the two integration plans; and a negative relationship between CSDC and horizontal integration (see Table 8).

The results suggest the following. First, the strongest motivation for integration or an M&A is EEUC perceptions. When there is external environment uncertainty, organizations tend to pool their efforts to overcome the difficulties. Second, counterintuitively, TDDC perceptions hinder integrations. Data, privacy, and intellectual property issues may be the underlying reasons for such findings. For example, Internet of Things (IoT) technology can bring in a privacy threat in the process of integration. There will be data acquisition, aggregation, and integration in an IoT ecosystem where different systems build the linkage [30]. Third, to provide better customer services, health systems may not pursue a vertical integration strategy (it is unclear in this study), but they will not pursue horizontal integration. Organizations may not think they can gain better customer services by acquiring a similar company.

**Table 8:** Summary of findings: relationship between competition and integration.

Variable	Vertical integration	Horizontal integration
EEUC^a^	Positive***	Positive***
TDDC^b^	Negative***	Negative*
CSDC^c^	N/A^d^	Negative***

^a^EEUC: environment uncertainty–related competition.

^b^TDDC: technology disruption–driven competition.

^c^CSDC: customer service–driven competition.

*** *P*<.001,* *P*<.05

### Implications

The findings of this study have several practical and policy implications. First, this study sheds light on the influence of the characteristics of health systems on competition perceptions. Medium size, major teaching status, high revenue, and having at least one uncompensated care burden hospital lead to lower perceptions of EEUC. Health systems with a high system-wide burden of uncompensated care feel a stronger sense of competition due to external environment uncertainty. Such differences support our definition and understanding of EEUC perceptions. In other words, external environment uncertainty is considered at the health-system level. Furthermore, the system-wide burden has an opposite influence on EEUC and TDDC. Health systems with a system-wide burden tend to perceive a higher level of external environment–driven competition but a lower level of technology-driven competition. This is a meaningful finding. The implication is that a system-wide burden motivates or even forces health systems to worry about external environment uncertainty, including changes in Medicare policy. Health systems with a system-wide uncompensated care burden may not think technological disruption can solve their problems. The main takeaway is that even in the post-COVID-19 era, where technologies such as telehealth dominate and change the health care industry, other organizational factors are still critical for competition.

Recent research provides a rationale in the technology context. For example, digital orientations of smaller and less complex health systems are not aspirational [31]. This study also implies that medium size, system-wide burden, and more hospitals in a system can have negative influences on TDDC, while health systems in the south and those with high revenue perceive TDDC negatively at an even higher level. In other words, health systems with such characteristics should leverage the technologies for their organizations. Furthermore, revenue from customer services may not be equally important for all health systems when examining the influence of CSDC. This supports the prior literature on competitive markets [32,33]. Thus, policies designed to reform the delivery system must consider the diversity in system characteristics [17].

Second, the critical implication of this study is related to how EEUC, TDDC, and CSDC perceptions influence vertical and horizontal integration plans by the leaders of health systems. We found that the EEUC perception is influential on both vertical and horizontal integration plans. This finding indicates that in unstable markets and situations such as the COVID-19–relevant financial disruptions, there may be more M&As. As a result, policymakers and practitioners may want to get ready for the changes in the post-COVID-19 new normal.

Regarding the influence of TDCC perceptions on vertical and horizontal integration, we found that technology-based competition drives less vertical integration. Given that health systems have recently aspired to accommodate remote- and virtual-care delivery options using technology, this is surprising. Plausibly, rather than acquiring firms, they may be focusing on developing in-house expertise. Indeed, a few telehealth M&As have been at the technology provider level rather than at the health system level. This may signal that health systems are preparing to develop technological expertise rather than making it an excuse for quick buyouts or mergers.

In addition, customer services should be an essential parameter for all health systems. However, whether it drives integration decisions remains an open question. We found that CSDC perceptions are not a strong driver of integration, although the external environment and technological uncertainties do affect horizontal integration. In other words, in a highly competitive market that includes external-, technological-, and customer service–relevant competitions, health systems will pursue horizontal integration strategies through mergers. This reflects an opportunistic behavior to monopolize markets and lock customers into one system.

There are a few other findings relevant to the contingent factors that influence integrations. For example, we found that health systems in specific regions and those with high revenue and high burden have a higher propensity for vertical integration, with slight variations in these findings for horizontal integration. We do not tease out many implications from these findings, except that they suggest that M&A decisions are influenced by a set of factors other than simply competition aspects.

There are strong indications that external uncertainty is a significant cause for thinking about integration. Perhaps policy interventions for fiscal assurance are a way to help suffering health systems survive the pandemic. The aspirations of some healthy and large health systems to take advantage of this situation to buy out weaker ones and emerge as the key dominant player in a region need to be kept in check.

### Limitations and Directions for Further Research

This study has a few limitations that future studies may be able to address. Focusing on internal issues (eg, management and coordination, value-chain dynamics) and demand-driven integration plans may be other perspectives to explore for health systems. Furthermore, opportunities for and barriers to integration may be studied in greater detail. Recent studies have shown that hospital M&As are associated with decreased patient experience measures, along with no evidence of quality improvement with the change in ownership of health entities [20]. Studies on the association between structural changes have yielded mixed results on cost, quality, and patient experience outcomes, indicating that the structural integration of health care organizations is conceptually distinct from integrated care delivery. Structural integration may or may not lead to integrated care, and providers and policymakers should focus on the conditions and strategies that enable structurally integrated organizations to capitalize on their ability to deliver more integrated care [16].

There are also concerns that integrated systems may increase prices and cost of care without commensurate improvements in quality and outcomes. The vertical integration approach showed no differences or lower efficiencies, as measured by utilization, spending, and prices [34]. Some evidence indicates that higher levels of integration are associated with higher levels of health care spending and increasing prices [35]. Other evidence shows mixed results, suggesting no significant effects or effects dependent on insurance type. While increased efficiencies may be possible, emerging research raises concerns about anticompetitive behavior, spending increases, and uncertain effects on quality. Vertical integration poses a threat to the affordability of health services and merits special attention from policymakers and antitrust authorities [13].

Reviews of hospital markets have found that concentrated markets are associated with higher hospital prices, with price increases often exceeding 20% after mergers. Of even more significant concern, reviews find that these price increases do not improve quality. In some cases, higher hospital concentration is associated with higher mortality rates. The higher concentration is associated with higher physician prices across a range of services. Despite evidence of associated price increases with no significant quality or efficiency improvements, both vertical and horizontal integration approaches have proceeded unchallenged. Health care consolidation concerns policymakers and regulators because market concentration can harm patients by increasing prices and premiums without accompanying improved care quality.

We also recognize that integration is not insufficient by itself, although we acknowledge that our underlying tone in this study has been to avoid overly integrated monopolistic endeavors. Future work might investigate M&As with outcomes, as done in prior work [9,26]. It is also worth noting that increases in the market concentration of health care providers and insurers have been examined nationally, and that increases in market concentration are associated with increases in prices and premiums. However, local markets for health care can differ dramatically. At the state level, laws and regulations, and the mix of providers and insurers, make markets in each state vastly different [36].

Another limitation of this study is that we used the cross-sectional data set to examine the relationships between variables. Future studies are planned to collect and use multiple years of data from health systems to address this limitation and provide causal inferences.

### Conclusions

Disruptions in health care are an emerging challenge and stem from a variety of sources. Recently, the COVID-19 pandemic has been a significant factor. In addition, technological disruptions and demands from empowered customers have put enormous pressure on health systems to shape different strategies for survival. Integration is one such strategy. However, understanding why leaders plan for integration is an essential insight for the health care sector. This study unravels competition-integration dynamics, and relates external environment uncertainty, technological competition, and customer services–driven competition to vertical and horizontal integration plans. Almost all health systems have some plans for integrations [37]; however, we found that environmental uncertainty drives integration more than other competitive factors. In addition, health systems with heavy competition dynamics will opt for mergers to alleviate survival challenges.

Competition-driven integration is unavoidable. However, overly integrated markets may lead to monopolistic entities and behavior, and that potential needs to be carefully managed and avoided with policy-level interventions. While interventions in the US health care sector are achieved through laws and regulations, proactively managing competition is an essential aspect of shaping policy interventions, and requires broader discussion and action.

## Appendix

Multimedia Appendix 1 Pairwise correlations among main variables (N=124).

## Abbreviations

AHRQ: Agency for Healthcare Research and Quality
CEO: chief executive officer
CSDC: customer service driven competition
EEUC: external environment uncertainty relevant competition
HARC: Health Administration Research Consortium
IoT: Internet of Things
HINT: horizontal integration
M&A: merger and acquisition
TDDC: technology disruption driven competition
VINT: vertical integration
