# Supplementary material for: Competition and Integration of US Health Systems in the Post-COVID-19 New Normal: Cross-sectional Survey
Source: JMIR Form Res. 2022 Mar 24;6(3):e32477. doi: 10.2196/32477 (PMC8954193; doi:10.2196/32477)
Supplement: Multimedia Appendix 1 [file formative_v6i3e32477_app1.docx]

**Multimedia Appendix 1. Pairwise correlations among main variables (N=124).**

| Variables^a^ | 1 | 2 | 3 | 4 | 5 | 6 | 7 | 8 | 9 | 10 | 11 | 12 | 13 | 14 | 15 | 16 | 17 | 18 | 19 | 20 |
| --- | --- | --- | --- | --- | --- | --- | --- | --- | --- | --- | --- | --- | --- | --- | --- | --- | --- | --- | --- | --- |
| 1. EEUC | 1.00 | 0.05 | 0.56 | 0.67 | 0.44 | –0.03 | –0.04 | –0.07 | –0.06 | 0.18 | 0.08 | –0.06 | –0.20 | –0.17 | –0.05 | –0.03 | 0.18– | –0.16 | –0.11 | –0.11 |
| 2. TDDC | 0.05 | 1.00 | 0.46 | –0.28 | –0.33 | –0.12 | 0.01 | –0.02 | 0.09 | 0.00 | –0.02 | 0.001 | 0.02 | 0.07 | 0.05 | 0.12 | –0.06 | –0.02 | –0.02 | 0.07 |
| 3. CSDC | 0.56 | 0.46 | 1.00 | 0.18 | –0.14 | –0.13 | 0.03 | –0.17 | 0.04 | 0.22 | 0.005 | 0.02 | –0.15 | –0.04 | –0.03 | 0.06 | –0.03 | –0.16 | –0.05 | 0.03 |
| 4. VINT | 0.67 | –0.28 | 0.18 | 1.00 | 0.82 | 0.08 | –0.04 | 0.02 | –0.003 | 0.18 | 0.03 | –0.08 | –0.06 | –0.09 | –0.06 | –0.09 | 0.18 | –0.12 | –0.09 | –0.12 |
| 5. HINT | 0.44 | –0.33 | –0.14 | 0.82 | 1.00 | 0.01 | 0.07 | 0.10 | –0.04 | 0.11 | –0.04 | –0.09 | 0.01 | –0.09 | 0.01 | –0.11 | 0.25 | –0.01 | 0.02 | –0.06 |
| 6. SIZE_B-MED. | –0.03 | –0.12 | –0.13 | 0.08 | 0.01 | 1.00 | –0.83 | –0.08 | –0.01 | –0.06 | 0.04 | –0.08 | –0.18 | –0.28 | –0.31 | –0.08 | –0.01 | –0.20 | –0.60 | –0.45 |
| 7. SIZE_B-LARGE | –0.04 | 0.01 | 0.03 | –0.04 | 0.07 | –0.83 | 1.00 | –0.02 | 0.11 | 0.02 | –0.14 | 0.25 | 0.27 | 0.37 | 0.37 | 0.17 | 0.05 | 0.27 | 0.75 | 0.52 |
| 8. REGION-MW | –0.07 | –0.02 | –0.17 | 0.02 | 0.10 | –0.08 | –0.02 | 1.00 | –0.42 | –0.26 | 0.07 | –0.002 | –0.13 | –0.10 | 0.09 | –0.20 | –0.09 | 0.01 | 0.01 | 0.03 |
| 9. REGN-SOUTH | –0.06 | 0.09 | 0.04 | –0.003 | –0.04 | –0.01 | 0.11 | –0.42 | 1.00 | –0.35 | 0.17 | 0.06 | 0.03 | 0.15 | –0.02 | 0.11 | 0.17 | 0.21 | 0.01 | 0.04 |
| 10. REGION-WEST | 0.18 | 0.00 | 0.22 | 0.18 | 0.11 | –0.06 | 0.02 | –0.26 | –0.35 | 1.00 | –0.06 | –0.03 | –0.01 | –0.03 | –0.02 | 0.15 | 0.07 | 0.01 | –0.02 | –0.04 |
| 11. TCHNG-MINOR | 0.08 | –0.02 | 0.005 | 0.03 | –0.04 | 0.04 | –0.14 | –0.07 | 0.17 | –0.06 | 1.00 | 0.01 | –0.07 | –0.07 | –0.05 | 0.04 | –0.06 | –0.08 | –0.06 | –0.08 |
| 12. TCHNG-MAJOR | –0.06 | 0.001 | 0.02 | –0.08 | –0.09 | –0.08 | 0.25 | –0.002 | 0.06 | –0.03 | 0.01 | 1.00 | –0.51 | 0.09 | 0.07 | 0.05 | –0.11 | 0.09 | 0.17 | 0.27 |
| 13. REV.-MED. | –0.20 | 0.02 | –0.15 | –0.06 | 0.01 | –0.18 | 0.27 | –0.13 | 0.03 | –0.01 | –0.07 | –0.51 | 1.00 | 0.17 | 0.15 | 0.33 | 0.01 | 0.10 | 0.40 | 0.04 |
| 14. REV.-HIGH | –0.17 | 0.07 | –0.04 | –0.09 | –0.09 | –0.28 | 0.37 | –0.10 | 0.15 | –0.03 | –0.07 | 0.09 | 0.17 | 1.00 | –0.23 | –0.05 | –0.05 | 0.12 | 0.28 | 0.26 |
| 15. HIGH-DSH-HOSP. | –0.05 | 0.05 | –0.03 | –0.06 | 0.01 | –0.31 | 0.37 | 0.09 | –0.02 | –0.02 | –0.05 | 0.07 | 0.15 | –0.23 | 1.00 | 0.14 | –0.03 | –0.01 | 0.50 | 0.30 |
| 16. HIGH-BURD.-SYS | –0.03 | 0.12 | 0.06 | –0.09 | –0.11 | –0.08 | 0.17 | –0.20 | 0.11 | 0.15 | 0.04 | 0.05 | 0.33 | –0.05 | 0.14 | 1.00 | –0.01 | 0.17 | 0.23 | 0.16 |
| 17. HIGH-BURD.-HOSP | 0.18 | –0.06 | –0.03 | 0.18 | 0.25 | –0.01 | 0.05 | –0.09 | 0.17 | 0.07 | –0.06 | –0.11 | 0.01 | –0.05 | –0.03 | –0.01 | 1.00 | 0.42 | –0.11 | –0.19 |
| 18. OWNERSHIP | –0.16 | –0.02 | –0.16 | –0.12 | –0.01 | –0.20 | 0.27 | 0.01 | 0.21 | 0.01 | –0.08 | 0.09 | 0.10 | 0.12 | –0.01 | 0.17 | 0.42 | 1.00 | 0.16 | 0.31 |
| 19. PHYSICIANS | –0.11 | –0.02 | –0.05 | –0.09 | 0.02 | –0.60 | 0.75 | 0.01 | 0.01 | –0.02 | –0.06 | 0.17 | 0.40 | 0.28 | 0.50 | 0.23 | –0.11 | 0.16 | 1.00 | 0.54 |
| 20. HOSPITALS | –0.11 | –0.07 | 0.03 | –0.12 | –0.06 | –0.45 | 0.52 | 0.03 | 0.04 | –0.04 | –0.08 | 0.27 | 0.04 | 0.26 | 0.30 | 0.16 | –0.19 | 0.31 | 0.54 | 1.00 |

^a^ See Table 2 for a description of variable codes.
